# Diet and general cognitive ability in the UK Biobank dataset

**DOI:** 10.1038/s41598-021-91259-3

**Published:** 2021-06-03

**Authors:** Piril Hepsomali, John A. Groeger

**Affiliations:** 1 Department of Psychology, University of Roehampton, London, UK; 2 Unilever R&D, Colworth Science Park, Bedford, UK; 3 Department of Psychology, Nottingham Trent University, School of Social Sciences, Nottingham, UK

**Keywords:** Psychology, Nutrition

## Abstract

Accumulating evidence suggests that dietary interventions might have potential to be used as a strategy to protect against age-related cognitive decline and neurodegeneration, as there are associations between some nutrients, food groups, dietary patterns, and some domains of cognition. In this study, we aimed to conduct the largest investigation of diet and cognition to date, through systematically examining the UK Biobank (UKB) data to find out whether dietary quality and food groups play a role on general cognitive ability. This cross-sectional population-based study involved 48,749 participants. UKB data on food frequency questionnaire and cognitive function were used. Also, healthy diet, partial fibre intake, and milk intake scores were calculated. Adjusted models included age, sex, and BMI. We observed associations between better general cognitive ability and higher intakes of fish, and unprocessed red meat; and moderate intakes of fibre, and milk. Surprisingly, we found that diet quality, vegetable intake, high and low fibre and milk intake were inversely associated with general cognitive ability. Our results suggest that fish and unprocessed red meat and/or nutrients that are found in fish and unprocessed red meat might be beneficial for general cognitive ability. However, results should be interpreted in caution as the same food groups may affect other domains of cognition or mental health differently. These discrepancies in the current state of evidence invites further research to examine domain-specific effects of dietary patterns/food groups on a wide range of cognitive and affective outcomes with a special focus on potential covariates that may have an impact on diet and cognition relationship.

## Introduction

It is expected that the prevalence of cognitive decline, ranging from age-related decline to dementia, will increase in coming years due to demographic aging^1^. Currently, it is known that over 50 million people worldwide suffer from Alzheimer’s disease, the most common type of dementia, and it is expected that this number will reach to 81.1 million people by 2040^2^ and 152 million by 2050^3^. These numbers imply remarkable economic and social burden for not only healthcare systems, but also for families, caregivers, and the elderly themselves. The current annual cost of dementia alone is estimated at US $1trillion, a figure set to double by 2030^3^. Given the expected increase in the number of dementia cases worldwide in the coming years and the impact of diet composition being the largest risk factor in neurological diseases^4^, developing nutritional interventions that are alternative or complementary to current treatments to protect against age-related cognitive decline and neurodegeneration is vital.

Mainly observational, but also some clinical trials have shown that various single nutrients (B vitamins, vitamin D, polyphenols, n-3 fatty acids), food groups (fish/seafood and vegetables), and dietary patterns (MED: Mediterranean, DASH: Dietary Approaches to Stop Hypertension, MIND: combination of MED and DASH) may protect against the development of age-related cognitive decline and pathological neurodegeneration via various mechanisms^5–7^. These mechanisms include, but not limited to (i) a reduction in neuroinflammation, (ii) an increase in endogenous antioxidant defence, and (iii) a modulation of the gut microbiota structure and function^5–7^. However, the findings to date are not very conclusive and they are heterogenous in terms of cognitive tests administered.

As nutritional research is shifting its direction from single nutrient or supplement analysis to food group and dietary pattern analysis^8^, it is crucial to understand the effect of food groups, diet quality/patterns, and the synergies and interactions between multiple nutrients and foods on general cognitive ability. Therefore, in the current study, our primary aim was to conduct the largest investigation to date, through systematic examination of the UK Biobank (UKB) data, to identify the associations of general cognitive ability with (i) the intake of food groups (vegetables, fruits, processed and unprocessed meat, fish), and (ii) diet quality/healthy diet score. Although not in line with the current shift of research focus towards food group and dietary pattern analysis, as the secondary objective of the current study, the associations between (i) fibre and (ii) milk intake and general cognitive ability were also examined in this large dataset to provide some further insights into this growing area of research.

## Method

This study was conducted using the UKB resource^9^. Ethical approval was granted by the North West Multi-Centre Ethics committee (Ref: 11/NW/0382). Re-analysis of UKB data under UKB project 61818 was performed in accordance with these guidelines and regulations, under the UKB ethics governance and framework (https://www.ukbiobank.ac.uk/ethics/).

### Study population

Detailed study design and methods of UKB have been reported elsewhere^9^. In brief, more than 500,000 eligible and consenting 40–69-year-old adults participated in the UKB trials between 2006 and 2010. At a baseline visit, after providing a written informed consent, participants completed a touch screen questionnaire that assessed various sociodemographic, lifestyle, and health behaviour variables, including diet and cognition.

### Measures

For the current study, we used the UKB Food Frequency questionnaire (FFQ) and calculated various diet-related scores (healthy diet score, partial fibre score and milk intake), where higher scores represent higher intakes. Briefly, UKB FFQ contains data on reported frequency of intake of a range of common food and drink items (https://biobank.ndph.ox.ac.uk/ukb/label.cgi?id=100052). Similar to our previous study^10^, as well as creating vegetable (UKB cooked + UKB salad/raw vegetable), fruit (UKB fresh + UKB dried fruit), unprocessed red meat (UKB beef + UKB lamb/mutton + UKB pork intake, fish (UKB oily + UKB non-oily fish) intake scores, we also utilised healthy diet score^11^, partial fibre score^12^ and milk intake^13^ calculations. Healthy diet score^11^ was calculated based on consumption of commonly eaten food groups following recommendations on dietary priorities for cardiometabolic health (Fruits: ≥ 3 servings/day, Vegetables: ≥ 3 servings/day, Fish: ≥ 2 servings/week, Processed meats: ≤ 1 serving/week, Unprocessed red meats: ≤ 1.5 servings/week, Whole grains: ≥ 3servings/day, Refined grains: ≤ 1.5 servings/day). Partial fibre score^12^ was obtained by (i) using the questions on fresh and dried fruit, raw/salad and cooked vegetables, bread type and intake, and breakfast cereal type and intake and (ii) assigning them portion sizes in grams, and (iii) multiplying the fibre content by the frequency of consumption. Milk intake^13^ was estimated by using the questions on main type of milk, bowls of breakfast cereal, cups of tea and coffee consumed and assigning them portion sizes in mL. For both partial fibre and milk intake scores, low, low/medium, medium, high/medium, and high intake groups were also created based on quintile splits.

In order to assess cognition, we used UKB’s cognitive function tests performed on the touchscreen questionnaire at baseline. For the current paper, we were interested in (1) UKB pairs matching (Field ID:399; number of errors made), (2) UKB reaction time (Field ID: 20023; mean time to correctly identify matches), (3) UKB prospective memory (Field ID: 20018; recall accuracy), (4) UKB fluid intelligence (Field ID: 20128; number of questions answered correctly in two minutes), and (5) UKB numeric memory (Field ID:4285; maximum number of digits remembered in reverse order) data fields to calculate a general cognitive ability score as per Fawns-Ritchie and Deary^14^. Details of the cognitive test battery and calculation of a general cognitive ability score based on five UKB cognitive battery tests are available elsewhere^15^. As per Fawns-Ritchie and Deary^14^, all five cognition measures (scores with non-normal distributions transformed accordingly) were entered into a Principle Components Analysis (PCA), and the scores on the first unrotated principal component were saved and used as general cognitive ability score, where higher scores represent better cognitive ability (i.e. increased speed and/or accuracy).

### Statistical analyses

All analyses were performed in IBM SPSS Statistics 26.0.0.0. Questionnaire response options, ‘do not know’ or ‘prefer not to answer’, were handled as missing values. PCA was used to calculate general cognitive ability scores. Separate unadjusted and adjusted (age, sex, and BMI) linear regressions were performed to examine the associations of food groups (vegetable, fruit, fish, unprocessed red meat, processed meat) with cognitive ability score. Separate univariate ANOVA’s and ANCOVA’s (adjusted for age, sex, BMI) were used to examine the effects of healthy diet score, partial fibre score, and milk intake on cognitive ability scores. Bonferroni post hoc test were used where appropriate. Results are reported following Publication Manual of the American Psychological Association (APA)^16^, where F refers to the F statistic, numbers in parentheses degrees of freedom regression and degrees of freedom error, and R2 and η2 reflect the variance accounted for and statistical power of the observed statistic, respectively.

## Results

### General cognitive ability

Unrotated loadings from the PCA using 5 UKB tests are reported in Table 1. Eigenvalues and scree plot indicated one component which accounted for 34% of the variance. Scores on the unrotated principal component were saved and used as a general cognitive ability score, where higher general cognitive ability scores represent better general cognitive ability.

**Table 1:** Loadings from a principal component analysis of 5 UK Biobank tests (*n* = 48,749). Eigenvalues and scree plot indicated one component. Unrotated findings are reported.

UKB cognitive test	Unrotated principle component 1
UKB pairs matching	− 0.46
UKB reaction time	− 0.44
UKB prospective memory	0.46
UKB fluid intelligence	0.74
UKB numeric memory	0.70
**Proportion of variance **	**0.34**

Of 48,749 participants, those who are male, younger, normal weight, most affluent, working (paid or unpaid), and who have higher education and income levels had better cognitive ability scores (Table 2 and Supplementary File Section 1 for detailed statistics including post hoc tests).

**Table 2:** Baseline characteristics of cognitive ability score (*n* = 48,749). Independent samples t-test for sex, and separate one-way ANOVAs for the remainder of the characteristics were conducted for the comparisons between participant characteristics and cognitive ability score.

Characteristics	*N*	Cognitive ability score *M* ± *SD*	p
**Sex**			< 0.0001
Male	26,623	0.09 ± 10.03	
Female	22,126	− 0.07 ± 0.96	
**Age**			< 0.0001
40–44	4998	0.38 ± 0.90	
45–49	5273	0.27 ± 0.87	
50–54	5838	0.23 ± 0.91	
55–59	6548	0.22 ± 0.87	
60–64	8303	0.10 ± 0.90	
65+	5368	− 0.10 ± 0.94	
**BMI**			< 0.0001
< 18.5 (underweight)	233	− 0.15 ± 1.00	
18.5–25 (normal)	16,179	0.08 ± 0.96	
25–30 (overweight)	20,435	− 0.003 ± 1.00	
30 + (obese)	11,737	− 0.09 ± 1.02	
**Townsend deprivation index quantiles**			< 0.0001
1 (most affluent)	6970	0.24 ± 0.86	
2	8474	0.22 ± 0.87	
3	8303	0.19 ± 0.90	
4	7538	0.17 ± 0.93	
5 (most deprived)	5043	− 0.24 ± 1.09	
**Employment status**			< 0.0001
In paid employment or self-employed	23,008	0.25 ± 0.89	
Retired	10,621	0.04 ± 0.91	
Looking after home and/or family	1008	0.18 ± 0.91	
Unable to work because of sickness or disability	780	− 0.22 ± 1.05	
Unemployed	653	0.08 ± 0.99	
Doing unpaid or voluntary work	145	0.24 ± 0.82	
Full-time or part-time student	113	0.18 ± 1.05	
**Qualifications**			< 0.0001
College or University Degree	14,319	0.41 ± 0.88	
A levels/AS levels or equivalent	4895	0.31 ± 0.85	
O levels/GCSEs or equivalent	9439	0.07 ± 0.85	
CSEs or equivalent	2535	− 0.26 ± 0.85	
NVQ or HND or HNC or equivalent	2911	− 0.26 ± 0.94	
Other professional qualifications	2229	− 0.12 ± 0.92	
**Total household income**			< 0.0001
Less than 18,000	6345	− 0.16 ± 0.98	
18,000–30,999	9380	0.03 ± 0.89	
31,000–51,999	10,617	0.24 ± 0.86	
52,000–100,000	8109	0.42 ± 0.83	
Greater than 100,000	1877	0.57 ± 0.86	

### Food groups and general cognitive ability

To quantify the associations between diet and cognitive performance, separate linear regression analyses were performed. In the model 1 (unadjusted), the association between food group intake (vegetable, fruit, fish, unprocessed red meat, processed meat) and cognitive ability score was analysed. In model 2, age, sex, and BMI were added as covariates in the linear model described in model 1. In both models, all the food groups examined were entered into the same model.

Results from statistical models are represented in Table 3. Model 1 revealed a statistically significant association between cognitive ability score and food group intake, *F*(5, 41,101) = 60.93, *p* < 0.0001, *R*^2^ = 0.007, with higher general cognitive ability score was associated with lower vegetable, fruit, unprocessed red meat intake, but higher processed meat intake. In age-, sex-, BMI-adjusted model 2, this association was significant again, *F*(8, 41,101) = 322.07, *p* < 0.0001, *R*^2^ = 0.059, however, fruit and processed meat intake were no longer associated with cognitive ability score, but lower intake of vegetables and higher intakes of fish and unprocessed red meat were associated with a better general cognitive ability score. For both models, multicollinearity was not detected (Tolerance range: 0.84–0.97; VIF range: 1.02–1.26).

**Table 3:** Regression analysis summary for general cognitive ability score.

Model	*B*	*SE*	*β*	*95% CI*	*p*
**1**					
(Constant)	0.170	0.018		[0.134, 0.206]	**0.000**
Vegetable intake	− 0.017	0.002	− 0.058	[− 0.020, − 0.014]	**0.000**
Fruit intake	− 0.012	0.002	− 0.029	[− 0.016, − 0.008]	**0.000**
Fish intake	− 0.004	0.003	− 0.006	[− 0.011, 0.002]	0.218
Unprocessed red meat intake	− 0.011	0.003	− 0.020	[− 0.017, − 0.005]	**0.000**
Processed meat intake	0.035	0.005	0.038	[0.026, 0.045]	**0.000**
**2**					
(Constant)	1.739	0.042		[1.657, 1.822]	**0.000**
Age	− 0.025	0.001	− 0.212	[− 0.026, − 0.024]	**0.000**
Sex (F = 0/M = 1)	0.162	0.010	0.083	[0.143, 0.181]	**0.000**
BMI	− 0.013	0.001	− 0.062	[− 0.015, − 0.011]	**0.000**
Vegetable intake	− 0.015	0.001	− 0.052	[− 0.018, − 0.013]	**0.000**
Fruit intake	− 0.002	0.002	− 0.006	[− 0.006, 0.002]	0.233
Fish intake	0.014	0.003	0.020	[0.007, 0.021]	**0.000**
Unprocessed red meat intake	0.002	0.003	0.004	[− 0.004, 0.008]	**0.006**
Processed meat intake	0.014	0.005	0.015	[0.004, 0.023]	0.485

In addition, please refer to supplementary material, Section 2.1, for the results of a further adjusted (qualifications and income) model that similarly showed associations between better general cognitive ability and (i) lower vegetable and fruit intake, and (ii) higher meat intake.

### Healthy diet, partial fibre, and milk intake scores

**Figure 1:**
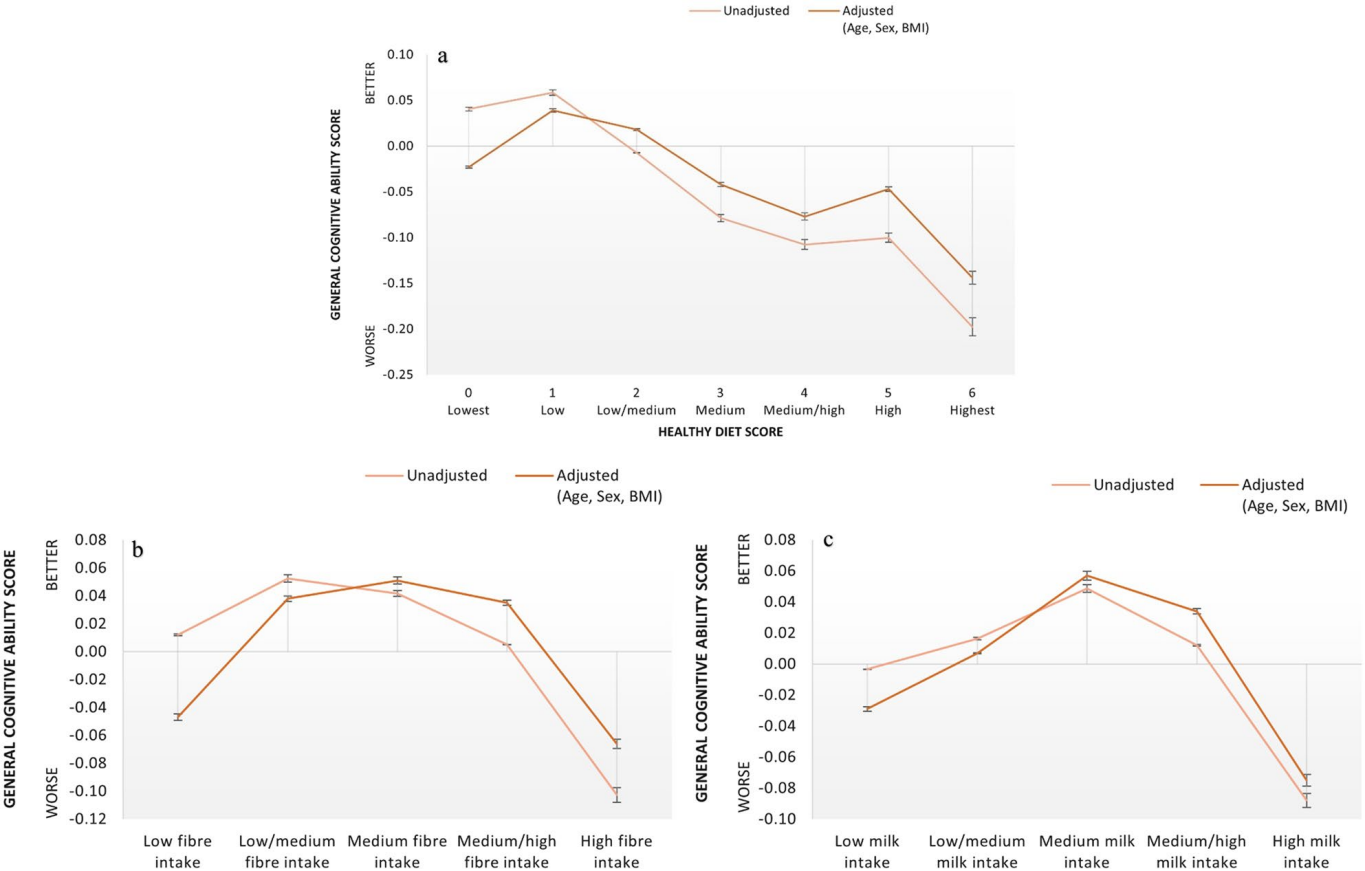
Mean cognitive ability scores according to (**a**) healthy diet scores, (**b**) fibre intake groups, (**c**) milk intake groups (bars represent 95% CI).

In order to examine the impact of healthy diet score (baseline characteristics of healthy diet score could be seen elsewhere^10^) on the general cognitive ability score, after excluding diet score of seven due to a low number of participants (n = 1) from the analyses, we conducted univariate ANOVA and ANCOVA (adjusted for age, sex, BMI) with healthy diet score as an independent variable and cognitive ability score as a dependent variable. We observed a significant main effect of healthy diet score on cognitive ability score, *F*(6, 46,625) = 31.68, *p* < 0.000, *η*^2^ = 0.004 (see Fig. 1a and Table 4). Lowest (0) and low (1) diet score groups had higher cognitive ability scores compared to low/medium (2), medium (3), medium/high (4) and high (5) diet score groups. Also, low/medium (2) diet score group had better cognitive performance compared to medium (3) and medium/high (4) diet score groups (see Table 5 for posthoc comparisons). In a significant adjusted model, *F*(6, 46,482) = 12.55, *p* < 0.000, *η*^2^ = 0.002, posthoc tests revealed that low (1) diet score group had better cognitive performance compared to the lowest (0) diet score group. Also, compared to low (1) and low/medium (2) diet score groups; medium (3) and medium/high(4) had worse cognitive performance (see Fig. 1a and Table 5 for posthoc comparisons). No other statistically significant differences were observed.

In addition, please refer to supplementary material, Section 2.2 for the results of a further adjusted (qualifications and income) model that showed consistent but slightly different results to age, sex, and BMI controlled models.

**Table 4:** Unadjusted and covariate adjusted descriptive statistics for cognitive ability scores across healthy diet score, partial fibre, and milk intake groups.

	Unadjusted		Adjusted	
	N	Mean ± SD	N	Mean_adj._ (SE)
**Healthy diet score**				
0 (Lowest)	6045	0.04 ± 1.00	6017	− 0.02 (0.01)
1 (Low)	15,950	0.06 ± 0.99	15,907	0.04 (0.01)
2 (Low/medium)	14,655	− 0.01 ± 0.99	14,614	0.02 (0.01)
3 (Medium)	8067	− 0.08 ± 1.00	8039	− 0.04 (0.01)
4 (Medium/high)	3188	− 0.11 ± 1.03	3168	− 0.08 (0.02)
5 (High)	750	− 0.10 ± 1.09	745	− 0.05 (0.04)
6 (Highest)	93	− 0.20 ± 1.03	93	− 0.14 (0.10)
**Partial fibre groups**				
Low	8795	0.01 ± 1.03	8760	− 0.05 (0.01)
Low/medium	9620	0.05 ± 0.98	9585	0.04 (0.01)
Medium	9832	0.04 ± 0.97	9805	0.05 (0.01)
Medium/high	10,151	0.01 ± 0.98	10,120	0.04 (0.01)
High	10,335	− 0.10 ± 1.03	10,298	− 0.07 (0.01)
**Milk intake groups**				
Low	8677	0.00 ± 1.04	8644	− 0.03 (0.01)
Low/medium	9809	0.02 ± 1.00	9774	0.01 (0.01)
Medium	9428	0.05 ± 0.97	9393	0.06 (0.01)
Medium/high	9729	0.01 ± 0.98	9702	0.03 (0.01)
High	9576	− 0.09 ± 1.01	9545	− 0.08 (0.01)

**Table 5:** Posthoc comparisons for unadjusted and covariate adjusted analyses using Bonferroni. Mean differences (columns-rows) shown. * shows mean difference is significant at the 0.05 level.

	Unadjusted							Adjusted						
	Healthy diet score							Healthy diet score						
	0	1	2	3	4	5	6	0	1	2	3	4	5	6
0 (Lowest)	1	− 0.02	0.05*	0.12*	0.15*	0.14*	0.24	1	− 0.06*	− 0.04	0.02	0.05	0.02	0.12
1 (Low)		1	0.07*	0.14*	0.17*	0.16*	0.26		1	0.02	0.08*	0.12*	0.09	0.18
2 (Low/medium)			1	0.07*	0.10*	0.09	0.19			1	0.06*	0.10*	0.06	0.16
3 (Medium)				1	0.03	0.02	0.12				1	0.04	0.01	0.10
4 (Medium/high)					1	− 0.01	0.09					1	− 0.03	0.07
5 (High)						1	0.10						1	0.10
6 (Highest)							1							1

	Partial fibre groups					Partial fibre groups								
	1	2	3	4	5	1	2	3	4	5				
1. Low	1	− 0.04	− 0.03	0.00	0.12*	1	− 0.09*	− 0.10*	− 0.08*	0.02				
2. Low/medium		1	0.01	0.05*	0.16*		1	− 0.01	0.00	0.10*				
3. Medium			1	0.04	0.14*			1	0.02	0.12*				
4. Medium/high				1	0.11*				1	0.10*				
5. High					1					1				

	Milk intake groups					Milk intake groups								
	1	2	3	4	5	1	2	3	4	5				
1. Low	1	− 0.02	− 0.05*	− 0.02	0.09*	1	− 0.04	− 0.09*	− 0.06*	0.05*				
2. Low/medium		1	− 0.03	0.00	0.10*		1	− 0.05*	− 0.03	0.08*				
3. Medium			1	0.04	0.14*			1	0.02	0.13*				
4. Medium/high				1	0.10*				1	0.11*				
5. High					**1**					**1**				

We explored the associations between fibre intake and cognitive ability by conducting univariate ANOVA and ANCOVA (adjusted for age, sex, BMI) tests with partial fibre score as an independent variable and cognitive ability score as a dependent variable, revealing a significant associations between partial fibre score on cognitive ability score, F (4, 46,613) = 38.10, p = 0.000, η^2^ = 0.003 in the unadjusted, and F (4, 46,469) = 24.39, p < 0.0001, η^2^ = 0.002 in the adjusted models (see Fig. 1b and Table 4). In the unadjusted model, the high fibre intake group had a lower general cognitive ability score compared to all other fibre intake groups, and the medium/high fibre intake group had a lower general cognitive ability score compared to the low/medium fibre intake group only. In the adjusted model, we observed an inverted U-shaped relationship with the high fibre intake group having worse cognitive performance compared to the low/medium, medium, and medium/high fibre intake groups. Also, the low fibre intake group performed worse than the low/medium, medium, and medium/high fibre intake groups (see Table 5 for posthoc comparisons). No other statistically significant differences were observed.

We also analysed the associations between milk intake and cognitive ability by using univariate ANOVA and ANCOVA (adjusted for age, sex, BMI) with milk intake as an independent variable, and cognitive ability score as a dependent variable. This analysis revealed significant associations between milk intake and cognitive ability score both in the unadjusted, F (4, 45,157) = 22.97, p = 0.000, η^2^  = 0.002, and adjusted models, F (4, 45,017) = 25.06, p = 0.000, η^2^ =  0.002 (see Fig. 1c and Table 4). In both unadjusted and adjusted analyses, the high milk intake group had a decreased general cognitive ability score compared to all other milk intake groups. In the unadjusted model, low milk intake group had lower general cognitive ability scores compared to medium milk intake group. In the adjusted model, on the other hand, both the medium and medium/high milk intake groups performed better than the low milk intake group. Additionally, the medium milk intake group also performed better than the low/medium milk intake group (see Table 5 for post hoc comparisons). No other statistically significant differences were observed.

Additionally, please refer to supplementary material, Sections 2.3 and 2.4 for the results of a further adjusted (qualifications and income) models for fibre and milk intake, respectively.

## Discussion and conclusion

In the current study, we examined the associations of food groups/items and healthy diet scores with general cognitive ability. After adjusting for age, sex, and BMI, we observed negative effects of higher vegetable intake and of healthy diet score on general cognitive ability. On the other hand, we showed the potential benefits of habitual consumption of fish and unprocessed red meat on general cognitive ability. Finally, we also showed inverted U-shaped relationships between milk and fibre intake; and general cognitive ability, where both lower and higher intakes of fibre and milk were associated with worse cognitive ability scores.

Unexpectedly, we found that higher vegetable intake was associated with worse general cognitive ability, but an association between fruit intake and general cognitive ability was lacking. Although these findings are not consistent with previous research showing positive effects of fruit and vegetable consumption, and/or adhering to diets in which these food groups are abundant, on cognition, see for a review^7^, they still could be explained based on the mixed effects of polyphenols on cognition. While some researchers observed a positive correlation between cognitive decline and the supplementation of different products rich in polyphenols on cognition, see for a review^5^, a recent meta-analysis concluded that some polyphenols might improve specific markers of cognitive status^17^, probably due to the (i) dual effects of polyphenols on the brain through prooxidant action^18^ and (ii) region-specific actions of polyphenols within brain structures^19^. Converging evidence comes from a study showing a positive association between high total polyphenol intake and language and verbal memory, but not with executive functioning; moreover, they also found negative associations between scores on executive functioning and intake of specific polyphenols including dihydrochalcones, catechins, proanthocyanins, and flavanols^20^. Similarly, a recent study also showed that adherence to the MED style diet (a diet rich in dietary polyphenols) was associated with better verbal ability but not with global cognitive function, visuospatial ability, and memory^21^. Given our general cognitive ability score is believed to measure processing speed, not non-speeded and verbal abilities^14^, these unexpected findings could be attributable to the lack of polyphenol effects on processing skills. Also, as our food group analysis approach might have failed to capture the interactive effects between multiple nutrients, food items and groups, the observed findings may simply reflect the simultaneous intake of another factor. Further research should examine the effects of various types and amounts of (i) polyphenol, and (ii) fruit and vegetable intake on various cognitive domains to uncover this complex and unexplained relationship.

Consistent with the results from previous reviews^7,22^, in the adjusted model, we showed the benefits of habitual consumption of fish and unprocessed red meat on general cognitive ability. As fish contains some essential macronutrients including protein and unsaturated fatty acids^23^ and as unprocessed red meat is a good source of protein, and B group vitamins^24^, our findings could be explained by the positive associations observed with cognition and (i) B vitamins, (ii) n−3 fatty acids, (iii) proteins, and (iv) dietary patterns that are moderately high in protein (e.g. DASH)^5,7,25,26^. Additionally, we observed a lack of association between processed meat intake and cognitive ability. Together these findings might reflect differential effects of protein quality (lean vs fatty) on cognition. Future research is warranted to examine the effects of not only protein quality (lean vs fatty), but also the source of protein (animal vs plant) on cognition. Specifically, exploration of potential neurocognitive effects of habitual consumption of non-animal sources protein (e.g. beans and pulses) is required. It is important to note that by using the same set of UKB cognition data, and analysing each component of cognitive ability score separately, Zhang and colleagues^27^ showed that red meat intake was negatively associated with UKB reaction time, UKB fluid intelligence, UKB numeric memory, and UKB prospective memory; but not with UKB pairs matching task. However, as they controlled for other variables including ethnicity, Townsend deprivation index, smoking, alcohol, education, physical activity level, sleeping hours, stroke history, and family history of dementia, general cognitive ability score might have been influenced differently by these covariates. Hence, more research is needed to explain our conflicting findings.

Although not consistent with previous research^5–7^, our finding showing a negative association between healthy diet score and general cognitive ability was not that surprising, given that (i) higher healthy diet scores represent higher intakes of vegetables, fruits, wholegrains; moderate intakes of fish; but lower intakes of processed, and unprocessed meat, and refined grains; (ii) we found negative associations between vegetable intake and general cognitive ability, and (iii) we found a positive association between unprocessed red meat intake. The discrepancy between previous findings and our results could be explained based on methodological differences. Firstly, it is possible that Lourida et al.’s^11^ healthy diet scores might not be applicable for multiple outcome variables, as different components of healthy diets might be beneficial to one aspect of mental health and wellbeing, but not another. For instance, by utilising the same healthy diet measure in another UKB study, we have shown associations between higher healthy diet scores and better sleep health and mental health^10^. However, in the current study we observed the opposite effect. Secondly, UKB’s FFQ did not measure nut, seed, legumes, and olive oil consumption, which are known to be some of the key elements of MED style diets^28^. Hence, the healthy diet score we utilised might not be sensitive enough to measure cognitive benefits associated with these crucial food items (and the whole MED diet). Thirdly, most of the studies that observed positive effects of MED on global cognition used well-known and validated measures of cognition (such as the Mini Mental State Examination…etc.), however, although validated in a previous study^14^, our general cognitive ability score might not (i) measure the same domains of cognition that the well-known global cognition tests measure and/or (ii) cover the full range of cognitive domains that the well-known global cognition tests cover. Finally, it is well known that not all cognitive domains are equally sensitive/responsive to food/diet^29^. Therefore, some, or all five, cognitive tasks included in the general cognitive ability score might not be sensitive enough to capture the effect of MED on global cognition. These methodological considerations highlight the importance and need for (i) creating well-defined/structured/validated healthy diet score/diet quality estimations, (ii) using validated cognitive tasks and (iii) identifying dietary components, and their mechanism of action, which might differentially affect different mental health and wellbeing components.

We observed a U-shaped relationship between cognitive ability and fibre intake, where lower and higher fibre intakes were associated with worse general cognitive ability. As low fibre intake also means lower intakes of vegetables, fruits, and wholegrains (hence lower healthy diet /Mediterranean diet scores) low fibre intake and worse general cognitive ability association is in line with previous studies showing negative associations between diet quality/health and cognition^5,7^. Given the role of dietary fibre consumption in lowering inflammation by modifying both the pH, and the permeability of the gut^30^; and the role of gut-brain-axis in cognition^31^; inflammation may be a potential mediator between the dietary fibre and the cognition curvilinear relationship observed here. In other words, moderate amounts of fibre may be beneficial for reducing or preventing inflammation (producing by-products which are beneficial for proper brain function), leading to an improvement in cognitive outcomes. Although supporting evidence comes from a review that highlighted the negative impact of higher levels of inflammation on cognitive processes, including memory, speed of processing, and global cognitive function^32^, future research is warranted to explain as to why high fibre intake might be detrimental to cognitive ability.

Milk intake and general cognitive ability also showed a curvilinear relationship, where both lower and higher milk intake were associated with lower general cognitive ability. As milk is a good source of protein and various group B vitamins, of especial importance for cognition^5,7^, it is clear why lower milk intake is associated with worse general cognitive ability. This finding is also in line with previous research showing associations between lower consumption of milk/dairy products and poor cognitive function, for a review see^33^. On the other hand, high milk intake and worse general cognitive ability association we observed, supporting previous findings that showed associations between (i) milk intake greater than 1 glass/day and greater cognitive decline^34^, (ii) whole-fat dairy intake and greater cognitive decline^33^, and (iii) saturated fat intakes from milk products and poorer global cognitive function and prospective memory^35^. Dietary saturated fatty acids^36^ and d-galactose^37,38^ in milk are known to cause systemic inflammation in humans and animals, respectively. Given the role of inflammation and the gut brain axis in modulating cognition^31,32,39^, these findings raise the possibility that higher milk intake, hence d-galactose and saturated fat intake, may cause inflammation and exacerbate cognition. Additionally, in the current study, we used Bradbury et al.’s^13^ milk intake estimation which is based on the questions on type of milk, bowls of breakfast cereal, cups of tea, and cups of coffee. Hence, these findings might also be partially attributable to the harmful effects of high doses of caffeine consumption on cognitive outcomes, or simply habituation, as higher doses might be required for habitual users to elicit better cognitive performance^40^.

It is also possible that, the associations with poor general cognitive ability and low (i) fibre, and (ii) milk intakes could be further explained by the impact of income on diet and dietary choices. Convergent evidence comes from observational studies that showed a negative impact of low income on diet quality, fibre intake, and milk intake^41–43^. Additionally, our supplementary analyses revealed that, after further controlling for income as well as qualifications, low partial fibre group did no longer have reduced general cognitive ability scores compared to low/medium and medium/high partial fibre intake groups, but this was not the case for milk intake (See Supplementary File Sections 2.3 and 2.4).

Although strengths and limitations of analysing UKB data, and adopting a fairly identical research approach as in the current study, were assessed elsewhere^10^, we would like to briefly mention the main limitation of this study. Unfortunately, we could not include total energy intake, an important confounder, in our analyses, due to unavailability of such data in the UKB FFQ. However, we controlled for BMI instead, which has been found to be a better estimate of objectively measured total energy expenditure (and therefore true energy intake) than estimated energy intake from a food-frequency questionnaire^44^. Also, our findings should be interpreted cautiously, as underlying cognitive deficits in the UKB participants might have negatively affected their dietary habits.

To sum up, in the current study, we observed positive associations between general cognitive ability and higher intakes of fish, unprocessed red meat, and moderate intakes of fibre and milk. Interestingly, higher intakes of vegetables and fruits (and higher healthy diet scores: where higher scores could be obtained by consuming higher amounts of vegetables, fruits, and wholegrains, but lower amounts of meat) were negatively associated with general cognitive ability. Although our findings emphasize the importance of nutrients derived from fish and unprocessed red meat for general cognitive ability, they also raise questions regarding the applicability of a ‘one-diet-cures-all’ approach, as higher vegetable, fruit, or fibre intake were found to be positively associated with better sleep and mental health in our earlier study^10^, but not with cognition in the current study. In other words, the same dietary patterns/food groups could affect health and wellbeing outcomes differently. Aside from the aforementioned suggestions, future research is needed to identify domain-specific effects of dietary patterns/food groups on a wide range of cognitive and affective outcomes.

In conclusion, the current research, along with our previous work^10^, highlight the importance of adhering to a balanced diet in order to maintain overall mental health and wellbeing, and we invite researchers and policymakers to target mental health and wellbeing outcomes as a whole when designing dietary interventions.

## Supplementary Information

Supplementary Information.

## Data Availability

The data that support the findings of this study are available from UK Biobank (http://www.ukbiobank.ac.uk/about-biobank-uk/). Restrictions apply to the availability of these data, which were used under license for the current study (Project ID: 61,818). Data are available for bona fide researchers upon application to the UK Biobank. For the current study, all relevant data are within the paper and the supplementary material.
