# Supplementary material for: Diet and general cognitive ability in the UK Biobank dataset
Source: Sci Rep. 2021 Jun 3;11:11786. doi: 10.1038/s41598-021-91259-3 (PMC8175590; doi:10.1038/s41598-021-91259-3)
Supplement: Supplementary file 1 — Supplementary Information. [file 41598_2021_91259_MOESM1_ESM.pdf]

Diet and general cognitive ability in the UK Biobank dataset

Piril Hepsomali[1,2*] & John A. Groeger[3]

[1] University of Roehampton, Department of Psychology, London, United Kingdom

[2] Unilever R&D, Colworth Science Park, Bedford, United Kingdom

[3]Nottingham Trent University, School of Social Sciences, Department of Psychology, Nottingham, United Kingdom

[*] Correspondence concerning this article should be addressed to Piril Hepsomali,

University of Roehampton, Department of Psychology, London, United Kingdom, email: P.Hepsomali@roehampton.ac.uk

Supplementary File

1. ANOVA and Bonferroni Post Hoc Tests for Baseline Characteristics

1.1. Age

	Sum of Squares	df	Mean Square	F	Sig.
Between Groups	2292.737	5	458.547	482.600	.000
Within Groups	46102.744	48521	.950		
Total	48395.481	48526			

		Mean Difference			95% Confidence Interval	
(I) age_cat	(J) age_cat	(I-J)	Std. Error	Sig.	Lower Bound	Upper Bound
40-44	45-49	.11193748*	.01809497	.000	.0588225	.1650524
	50-54	.18765688*	.01741741	.000	.1365308	.2387830
	55-59	.22396971*	.01675627	.000	.1747843	.2731551
	60-64	.41744937*	.01568988	.000	.3713942	.4635046
	65+	.67604921*	.01657124	.000	.6274069	.7246915
45-49	40-44	-.11193748*	.01809497	.000	-.1650524	-.0588225
	50-54	.07571940*	.01710726	.000	.0255037	.1259351
	55-59	.11203223*	.01643365	.000	.0637938	.1602707
	60-64	.30551189*	.01534486	.000	.2604694	.3505543
	65+	.56411173*	.01624495	.000	.5164272	.6117963
50-54	40-44	-.18765688*	.01741741	.000	-.2387830	-.1365308
	45-49	-.07571940*	.01710726	.000	-.1259351	-.0255037
	55-59	.03631283	.01568449	.309	-.0097265	.0823522
	60-64	.22979249*	.01453970	.000	.1871134	.2724715
	65+	.48839233*	.01548666	.000	.4429337	.5338510
55-59	40-44	-.22396971*	.01675627	.000	-.2731551	-.1747843
	45-49	-.11203223*	.01643365	.000	-.1602707	-.0637938
	50-54	-.03631283	.01568449	.309	-.0823522	.0097265
	60-64	.19347966*	.01374079	.000	.1531457	.2338136
	65+	.45207950*	.01473916	.000	.4088150	.4953440
60-64	40-44	-.41744937*	.01568988	.000	-.4635046	-.3713942
	45-49	-.30551189*	.01534486	.000	-.3505543	-.2604694
	50-54	-.22979249*	.01453970	.000	-.2724715	-.1871134
	55-59	-.19347966*	.01374079	.000	-.2338136	-.1531457
	65+	.25859984*	.01351454	.000	.2189300	.2982697
65+	40-44	-.67604921*	.01657124	.000	-.7246915	-.6274069
	45-49	-.56411173*	.01624495	.000	-.6117963	-.5164272
	50-54	-.48839233*	.01548666	.000	-.5338510	-.4429337
	55-59	-.45207950*	.01473916	.000	-.4953440	-.4088150
	60-64	-.25859984*	.01351454	.000	-.2982697	-.2189300

*. The mean difference is significant at the 0.05 level.

1.2. BMI

	Sum of Squares	df	Mean Square	F	Sig.
Between Groups	218.335	3	72.778	73.306	.000
Within Groups	48230.008	48580	.993		
Total	48448.343	48583			

(I) BMIcat	(J) BMIcat	Mean Difference (I-J)	Std. Error	Sig.	95% Confidence Interval Lower Bound	95% Confidence Interval Upper Bound
<18.5	18.5-25	-.23419018*	.06574411	.002	-.4076472	-.0607332
	25-30	-.14825920	.06564684	.144	-.3214596	.0249412
	30+	-.05802860	.06592050	1.000	-.2319510	.1158938
18.5-25	<18.5	.23419018*	.06574411	.002	.0607332	.4076472
	25-30	.08593098*	.01048553	.000	.0582663	.1135956
	30+	.17616158*	.01208098	.000	.1442875	.2080356
25-30	<18.5	.14825920	.06564684	.144	-.0249412	.3214596
	18.5-25	-.08593098*	.01048553	.000	-.1135956	-.0582663
	30+	.09023060*	.01153993	.000	.0597841	.1206771
30+	<18.5	.05802860	.06592050	1.000	-.1158938	.2319510
	18.5-25	-.17616158*	.01208098	.000	-.2080356	-.1442875
	25-30	-.09023060*	.01153993	.000	-.1206771	-.0597841

*. The mean difference is significant at the 0.05 level.

1.3. Deprivation

	Sum of Squares	df	Mean Square	F	Sig.
Between Groups	757.401	4	189.350	192.351	.000
Within Groups	47864.597	48623	.984		
Total	48621.998	48627			

(I) Percentile Group of Deprivation_Index	(J) Percentile Group of Deprivation_Index	Mean Difference (I-J)	Std. Error	Sig.	95% Confidence Interval	
					Lower Bound	Upper Bound
1 (most affluent)	2	.05272512*	.01418897	.002	.0128944	.0925559
	3	.10472984*	.01417646	.000	.0649342	.1445255
	4	.13876536*	.01447582	.000	.0981294	.1794013
	5	.39467813*	.01551672	.000	.3511202	.4382361
2	1	-.05272512*	.01418897	.002	-.0925559	-.0128944
	3	.05200472*	.01334875	.001	.0145326	.0894768
	4	.08604024*	.01366626	.000	.0476768	.1244036
	5	.34195301*	.01476434	.000	.3005071	.3833989
3	1	-.10472984*	.01417646	.000	-.1445255	-.0649342
	2	-.05200472*	.01334875	.001	-.0894768	-.0145326
	4	.03403552	.01365327	.127	-.0042914	.0723625
	5	.28994829*	.01475232	.000	.2485361	.3313604
4	1	-.13876536*	.01447582	.000	-.1794013	-.0981294
	2	-.08604024*	.01366626	.000	-.1244036	-.0476768
	3	-.03403552	.01365327	.127	-.0723625	.0042914
	5	.25591277*	.01504022	.000	.2136924	.2981331
5 (least affluent)	1	-.39467813*	.01551672	.000	-.4382361	-.3511202
	2	-.34195301*	.01476434	.000	-.3833989	-.3005071
	3	-.28994829*	.01475232	.000	-.3313604	-.2485361
	4	-.25591277*	.01504022	.000	-.2981331	-.2136924

*. The mean difference is significant at the 0.05 level.

1.4. Employment

	Sum of Squares	df	Mean Square	F	Sig.
Between Groups	1833.956	6	305.659	318.502	.000
Within Groups	46402.331	48352	.960		
Total	48236.288	48358			

(I) current_employment_status	(J) current_employment_status	Mean Difference (I-J)	Std. Error	Sig.	95% Confidence Interval Lower Bound	Upper Bound
In paid employment or self-employed	retired	.37155279*	.00959198	.000	.3424101	.4006955
	Looking after home and/or family	.18469013*	.02629452	.000	.1048012	.2645791
	Unable to work because of sickness or disability	.67742166*	.02697954	.000	.5954515	.7593918
	unemployed	.24628006*	.03313236	.000	.1456161	.3469440
	Doing unpaid or voluntary work	.14650350	.06785927	.648	-.0596689	.3526759
	full_part_time_student	.13946858	.08330152	1.000	-.1136210	.3925582
retired	In paid employment or self-employed	-.37155279*	.00959198	.000	-.4006955	-.3424101
	Looking after home and/or family	-.18686266*	.02671016	.000	-.2680144	-.1057109
	Unable to work because of sickness or disability	.30586887*	.02738479	.000	.2226675	.3890703
	unemployed	-.12527273*	.03346318	.004	-.2269417	-.0236037
	Doing unpaid or voluntary work	-.22504929*	.06802141	.020	-.4317143	-.0183843
	full_part_time_student	-.23208421	.08343365	.114	-.4855752	.0214068
Looking after home and/or family	In paid employment or self-employed	-.18469013*	.02629452	.000	-.2645791	-.1048012
	retired	.18686266*	.02671016	.000	.1057109	.2680144
	Unable to work because of sickness or disability	.49273153*	.03673312	.000	.3811277	.6043354
	unemployed	.06158993	.04146300	1.000	-.0643844	.1875643
	Doing unpaid or voluntary work	-.03818663	.07229321	1.000	-.2578304	.1814571
	full_part_time_student	-.04522155	.08695154	1.000	-.3094007	.2189577
Unable to work because of sickness or disability	In paid employment or self-employed	-.67742166*	.02697954	.000	-.7593918	-.5954515
	retired	-.30586887*	.02738479	.000	-.3890703	-.2226675
	Looking after home and/or family	-.49273153*	.03673312	.000	-.6043354	-.3811277
	unemployed	-.43114160*	.04190076	.000	-.5584460	-.3038372
	Doing unpaid or voluntary work	-.53091815*	.07254517	.000	-.7513274	-.3105089
	full_part_time_student	-.53795307*	.08716113	.000	-.8027691	-.2731371
unemployed	In paid employment or self-employed	-.24628006*	.03313236	.000	-.3469440	-.1456161

	retired	.12527273*	.03346318	.004	.0236037	.2269417
	Looking after home and/or family	-.06158993	.04146300	1.000	-.1875643	.0643844
	Unable to work because of sickness or disability	.43114160*	.04190076	.000	.3038372	.5584460
	Doing unpaid or voluntary work	-.09977656	.07505104	1.000	-.3277993	.1282462
	full_part_time_student	-.10681148	.08925761	1.000	-.3779971	.1643741
Doing unpaid or voluntary work	In paid employment or self-employed	-.14650350	.06785927	.648	-.3526759	.0596689
	retired	.22504929*	.06802141	.020	.0183843	.4317143
	Looking after home and/or family	.03818663	.07229321	1.000	-.1814571	.2578304
	Unable to work because of sickness or disability	.53091815*	.07254517	.000	.3105089	.7513274
	unemployed	.09977656	.07505104	1.000	-.1282462	.3277993
	full_part_time_student	-.00703492	.10711699	1.000	-.3324815	.3184117
full_part_time_student	In paid employment or self-employed	-.13946858	.08330152	1.000	-.3925582	.1136210
	retired	.23208421	.08343365	.114	-.0214068	.4855752
	Looking after home and/or family	.04522155	.08695154	1.000	-.2189577	.3094007
	Unable to work because of sickness or disability	.53795307*	.08716113	.000	.2731371	.8027691
	unemployed	.10681148	.08925761	1.000	-.1643741	.3779971
	Doing unpaid or voluntary work	.00703492	.10711699	1.000	-.3184117	.3324815

*. The mean difference is significant at the 0.05 level.

1.5. Qualifications

	Sum of Squares	df	Mean Square	F	Sig.
Between Groups	2736.338	5	547.268	689.049	.000
Within Groups	32759.860	41247	.794		
Total	35496.199	41252			

					95% Confidence Interval	
(I) qualifications	(J) qualifications	Mean Difference (I-J)	Std. Error	Sig.	Lower Bound	Upper Bound
College or University degree	A levels/AS levels or equivalent	.10236208*	.01395588	.000	.0613964	.1433278
	O levels/GCSEs or equivalent	.34869429*	.01107412	.000	.3161876	.3812009
	CSEs or equivalent	.69378482*	.01785222	.000	.6413820	.7461877
	NVQ or HND or HNC or equivalent	.69979891*	.01686505	.000	.6502937	.7493041
	Other professional qualifications eg: nursing, teaching	.56123816*	.01862112	.000	.5065783	.6158981
A levels/AS levels or equivalent	College or University degree	-.10236208*	.01395588	.000	-.1433278	-.0613964
	O levels/GCSEs or equivalent	.24633221*	.01468811	.000	.2032172	.2894473
	CSEs or equivalent	.59142274*	.02029301	.000	.5318553	.6509902
	NVQ or HND or HNC or equivalent	.59743682*	.01943024	.000	.5404019	.6544718
	Other professional qualifications eg: nursing, teaching	.45887608*	.02097262	.000	.3973137	.5204385
O levels/GCSEs or equivalent	College or University degree	-.34869429*	.01107412	.000	-.3812009	-.3161876
	A levels/AS levels or equivalent	-.24633221*	.01468811	.000	-.2894473	-.2032172
	CSEs or equivalent	.34509053*	.01843029	.000	.2909908	.3991903
	NVQ or HND or HNC or equivalent	.35110461*	.01747581	.000	.2998066	.4024026
	Other professional qualifications eg: nursing, teaching	.21254387*	.01917603	.000	.1562551	.2688326
CSEs or equivalent	College or University degree	-.69378482*	.01785222	.000	-.7461877	-.6413820

	A levels/AS levels or equivalent	-.59142274*	.02029301	.000	-.6509902	-.5318553
	O levels/GCSEs or equivalent	-.34509053*	.01843029	.000	-.3991903	-.2909908
	NVQ or HND or HNC or equivalent	.00601409	.02239351	1.000	-.0597192	.0717473
	Other professional qualifications eg: nursing, teaching	-.13254666*	.02374417	.000	-.2022446	-.0628487
NVQ or HND or HNC or equivalent	College or University degree	-.69979891*	.01686505	.000	-.7493041	-.6502937
	A levels/AS levels or equivalent	-.59743682*	.01943024	.000	-.6544718	-.5404019
	O levels/GCSEs or equivalent	-.35110461*	.01747581	.000	-.4024026	-.2998066
	CSEs or equivalent	-.00601409	.02239351	1.000	-.0717473	.0597192
	Other professional qualifications eg: nursing, teaching	-.13856075*	.02301117	.000	-.2061071	-.0710144
Other professional qualifications eg: nursing, teaching	College or University degree	-.56123816*	.01862112	.000	-.6158981	-.5065783
	A levels/AS levels or equivalent	-.45887608*	.02097262	.000	-.5204385	-.3973137
	O levels/GCSEs or equivalent	-.21254387*	.01917603	.000	-.2688326	-.1562551
	CSEs or equivalent	.13254666*	.02374417	.000	.0628487	.2022446
	NVQ or HND or HNC or equivalent	.13856075*	.02301117	.000	.0710144	.2061071

*. The mean difference is significant at the 0.05 level.

1.6. Income

	Sum of Squares	df	Mean Square	F	Sig.
Between Groups	3733.312	4	933.328	1079.785	.000
Within Groups	36579.055	42319	.864		
Total	40312.367	42323			

(I) total_household _income	(J) total_household_income	Mean Difference (I-J)	Std. Error	Sig.	95% Confidence Interval	
					Lower Bound	Upper Bound
Less than 18,000	18,000 to 30,999	-.31202669*	.01300009	.000	-.3485203	-.2755331
	31,000 to 51,999	-.58781591*	.01293853	.000	-.6241367	-.5514951
	52,000 to 100,000	-.78680087*	.01394462	.000	-.8259459	-.7476558
	Greater than 100,000	-.93084568*	.02325550	.000	-.9961281	-.8655633
18,000 to 30,999	Less than 18,000	.31202669*	.01300009	.000	.2755331	.3485203
	31,000 to 51,999	-.27578921*	.01238061	.000	-.3105438	-.2410346
	52,000 to 100,000	-.47477418*	.01342857	.000	-.5124706	-.4370778
	Greater than 100,000	-.61881899*	.02294978	.000	-.6832432	-.5543948
31,000 to 51,999	Less than 18,000	.58781591*	.01293853	.000	.5514951	.6241367
	18,000 to 30,999	.27578921*	.01238061	.000	.2410346	.3105438
	52,000 to 100,000	-.19898497*	.01336898	.000	-.2365141	-.1614558
	Greater than 100,000	-.34302978*	.02291496	.000	-.4073562	-.2787033
52,000 to 100,000	Less than 18,000	.78680087*	.01394462	.000	.7476558	.8259459
	18,000 to 30,999	.47477418*	.01342857	.000	.4370778	.5124706
	31,000 to 51,999	.19898497*	.01336898	.000	.1614558	.2365141
	Greater than 100,000	-.14404481*	.02349771	.000	-.2100071	-.0780825
Greater than 100,000	Less than 18,000	.93084568*	.02325550	.000	.8655633	.9961281
	18,000 to 30,999	.61881899*	.02294978	.000	.5543948	.6832432
	31,000 to 51,999	.34302978*	.02291496	.000	.2787033	.4073562
	52,000 to 100,000	.14404481*	.02349771	.000	.0780825	.2100071

*. The mean difference is significant at the 0.05 level.

2. Further Controlling for Qualifications and Income

To quantify the associations between diet and cognitive performance, a linear regression analysis (further adjusted by qualifications and income) was performed. We also conducted separate univariate ANCOVA tests (further adjusted by qualifications and income) on general cognitive ability and (i) partial fibre intake, and (ii) milk intake associations.

2.1. Regression Analysis for General Cognitive Ability Score (Model 3)

Table 2.1.1. Regression Analysis for General Cognitive Ability Score (further adjusted for qualifications and income)

	B	*SE*	*β*	*95% CI*	*p*
(Constant)	0.784	0.047		[0.692, 0.876]	0.000
Age	-0.009	0.001	-0.084	[-0.010, -0.008]	0.000
Sex (F=0/M=1)	0.134	0.010	0.073	[0.115, 0.153]	0.000
BMI	-0.003	0.001	-0.017	[-0.005, -0.001]	0.001
Qualifications	-0.125	0.003	-0.213	[-0.131, -0.119]	0.000
Income	0.124	0.004	0.156	[0.116, 0.133]	0.000
Vegetable intake	-0.015	0.001	-0.054	[-0.018, -0.012]	0.000
Fruit intake	-0.012	0.002	-0.033	[-0.016, -0.008]	0.000
Fish intake	-0.002	0.003	-0.004	[-0.009, 0.004]	0.487
Unprocessed red meat intake	0.011	0.003	0.021	[0.005, 0.017]	0.000
Processed meat intake	0.038	0.005	0.044	[0.028, 0.047]	0.000

$F(10, 35712)=$ 498.18, $p<.0001$, $R^2= .122$
Tolerance range: 0.78 – 0.96; VIF range: 1.04- 1.29

2.2. Impact of Qualifications and Income on Healthy Diet Score and General Cognitive Ability Association

After further adjustments, ANCOVA revealed a significant association between healthy diet score and general cognitive ability, $F(6, 36595)= 21.94$, $p<.001$, $\eta 2= .004$ (please see (i) Table 2.2.1 for means(se), (ii) Table 2.2.2 for post hoc test results, and (iii) Supplementary Figure 1A for aa graphical representation of the results).

Table 2.2.1. General Cognitive Ability Score across Healthy Diet Scores (further adjusted for qualifications and income)

Healthy Diet Score	*N*	*Mean* $_{adj.}$ *(SE)*
0 (Lowest)	4394	0.23 (0.01)
1 (Low)	12098	0.22 (0.01)
2 (Low/medium)	11001	0.18 (0.01)
3 (Medium)	6082	0.11 (0.01)
4 (Medium/high)	2406	0.07 (0.02)
5 (High)	555	0.12 (0.04)
6 (Highest)	71	-0.05 (0.10)

Table 2.2.2. Posthoc Comparisons for Covariate Adjusted Analyses Using Bonferroni. Mean differences (column-rows) shown. * shows mean difference is significant at the 0.05 level.

	Healthy Diet Score						
	0	1	2	3	4	5	6
0 (Lowest)	1	.01	.06*	.13*	.16*	.11	.28
1 (Low)		1	.47*	.12	.12*	.10	.27
2 (Low/medium)			1	.07*	.10*	.06	.22
3 (Medium)				1	.03	-.01	.15
4 (Medium/high)					1	-.05	.12
5 (High)						1	.16
6 (Highest)							1

2.3. Impact of Qualifications and Income on Partial Fibre Score and General Cognitive Ability Association

The association between partial fibre intake score and general cognitive ability remained significant after further controlling for qualifications and income, $F(4, 36601)= 22.19$, $p<.001$, $\eta 2= .002$ (please see (i) Table 2.3.1 for means(se), (ii) Table 2.3.2 for post hoc test results, and (iii) Supplementary Figure 1B for aa graphical representation of the results).

Table 2.3.1. General Cognitive Ability Score across Partial Fibre Intake Groups (further adjusted for qualifications and income)

Partial Fibre Intake Groups	*N*	*Mean* $_{adj.}$ *(SE)*
Low	6456	0.20 (0.01)
Low/medium	7392	0.20 (0.01)
Medium	7484	0.21 (0.01)
Medium/high	7677	0.19 (0.01)
High	7592	0.10 (0.01)

Table 2.3.2. Posthoc Comparisons Adjusted (Groups (further adjusted for qualifications and income) Analyses Using Bonferroni. Mean differences (column-rows) shown. * shows mean difference is significant at the 0.05 level.

	Partial Fibre Groups				
	1	2	3	4	5
1. Low	1	-.01	-.02	.01	.10*
2. Low/medium		1	-.01	.01	.11*
3. Medium			1	.03	.12*
4. Medium/high				1	.09*
5. High					1

2.4. Impact of Qualifications and Income on Milk Intake and General Cognitive Ability Association

This analysis also revealed a significant association between milk intake and general cognitive ability, $F(4, 35424)= 7.45$, $p<.001$, $\eta 2= .001$ (please see (i) Table 2.4.1 for means(se), (ii) Table 2.4.2 for post hoc test results, and (iii) Supplementary Figure 1C for aa graphical representation of the results).

Table 2.4.1. General Cognitive Ability Score across Milk Intake Groups (further adjusted for qualifications and income)

Milk Intake Groups	*N*	*Mean adj. (SE)*
Low	6587	0.16 (0.01)
Low/medium	7423	0.16 (0.01)
Medium	7203	0.21 (0.01)
Medium/high	7292	0.20 (0.01)
High	6920	0.15 (0.01)

Table 2.4.2. Posthoc Comparisons Adjusted (Groups (further adjusted for qualifications and income) Analyses Using Bonferroni. Mean differences (column-rows) shown. * shows mean difference is significant at the 0.05 level.

	Milk Intake Groups				
	1	2	3	4	5
1. Low	1	-.01	-.06*	-.04*	.01
2. Low/medium		1	-.05*	-.03	.01
3. Medium			1	.01	.06*
4. Medium/high				1	.05*
5. High					1

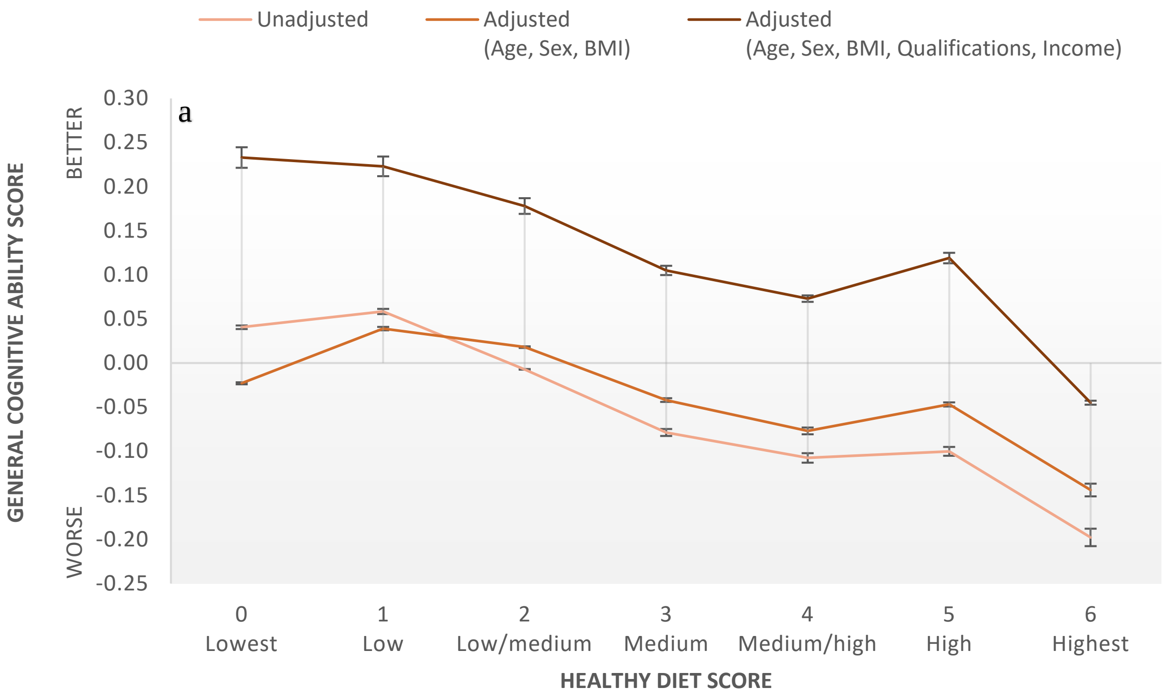
Unadjusted
Adjusted
(Age, Sex, BMI)
Adjusted
(Age, Sex, BMI, Qualifications, Income)
a
GENERAL COGNITIVE ABILITY SCORE
BETTER
WORSE
0.30
0.25
0.20
0.15
0.10
0.05
0.00
-0.05
-0.10
-0.15
-0.20
-0.25
0
Lowest
1
Low
2
Low/medium
3
Medium
4
Medium/high
5
High
6
Highest
HEALTHY DIET SCORE

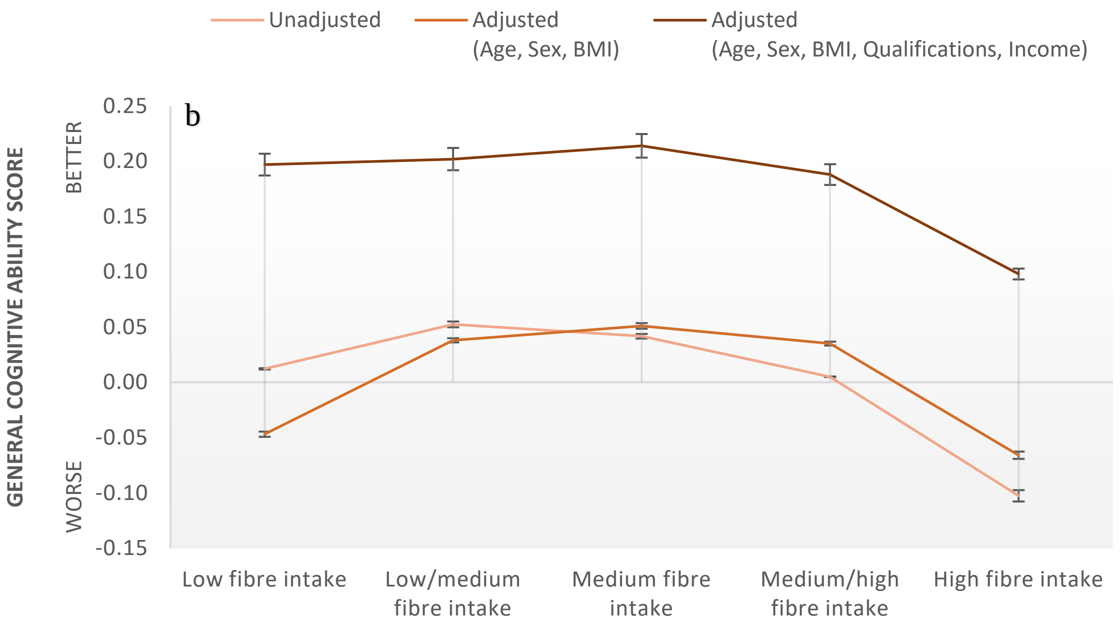
Unadjusted
Adjusted
(Age, Sex, BMI)
Adjusted
(Age, Sex, BMI, Qualifications, Income)
b
GENERAL COGNITIVE ABILITY SCORE
BETTER
WORSE
0.25
0.20
0.15
0.10
0.05
0.00
-0.05
-0.10
-0.15
Low fibre intake
Low/medium fibre intake
Medium fibre intake
Medium/high fibre intake
High fibre intake

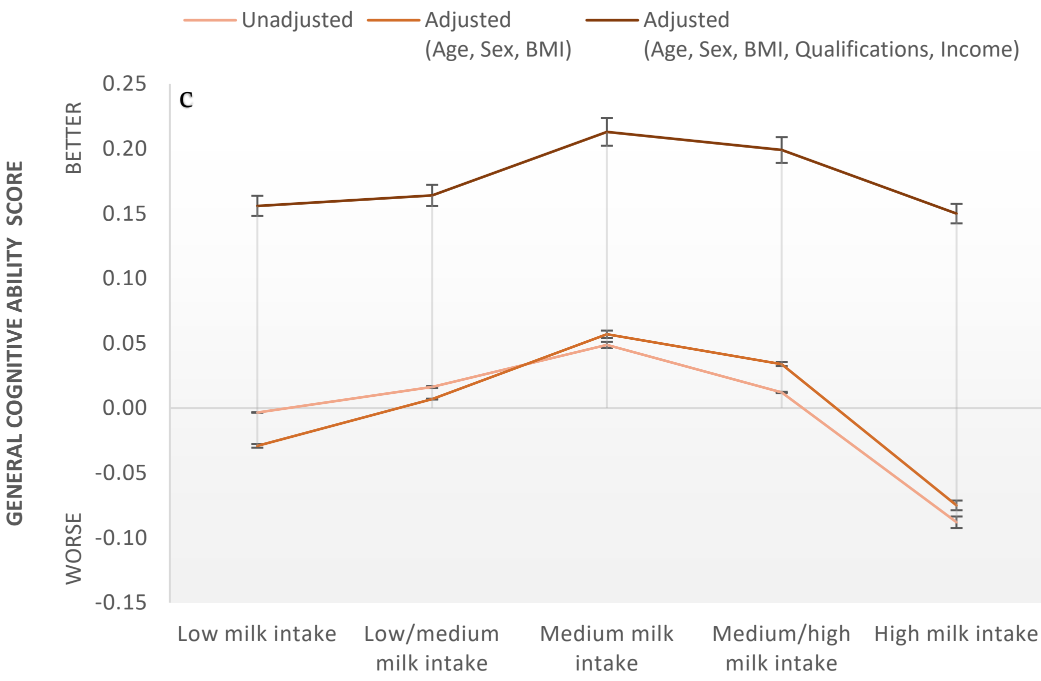

Supplementary Figure 1. Mean cognitive ability scores according to (a) healthy diet scores, (b) fibre intake groups, (c) milk intake groups (bars represent 95% CI).
